# Bacterial Ribosomes Induce Plasticity in Mouse Adult Fibroblasts

**DOI:** 10.3390/cells13131116

**Published:** 2024-06-27

**Authors:** Anamika Datta, Arif Istiaq, Shigehiko Tamura, Kunimasa Ohta

**Affiliations:** 1 Department of Stem Cell Biology, Graduate School of Systems Life Sciences, Kyushu University, Fukuoka 819-0395, Japan; datta.anamika.295@s.kyushu-u.ac.jp; 2 Department of Stem Cell Biology, Faculty of Arts and Science, Kyushu University, Fukuoka 819-0395, Japan; arifi@wustl.edu; 3 Department of Pediatrics, Washington University School of Medicine, St. Louis, MO 63110-1010, USA; 4 Department of Molecular Cell Biology, Faculty of Arts and Science, Kyushu University, Fukuoka 819-0395, Japan; stamura@artsci.kyushu-u.ac.jp

**Keywords:** ribosome, plasticity, lineage conversion, mouse adult fibroblasts, non-canonical, multipotency

## Abstract

The incorporation of bacterial ribosome has been reported to induce multipotency in somatic and cancer cells which leads to the conversion of cell lineages. Queried on its universality, we observed that bacterial ribosome incorporation into trypsinized mouse adult fibroblast cells (MAF) led to the formation of ribosome-induced cell clusters (RICs) that showed strong positive alkaline phosphatase staining. Under in vitro differentiation conditions, RICs-MAF were differentiated into adipocytes, osteoblasts, and chondrocytes. In addition, RICs-MAF were able to differentiate into neural cells. Furthermore, RICs-MAF expressed early senescence markers without cell death. Strikingly, no noticeable expression of renowned stemness markers like Oct4, Nanog, Sox2, etc. was observed here. Later RNA-sequencing data revealed the expression of rare pluripotency-associated markers, i.e., Dnmt3l, Sox5, Tbx3 and Cdc73 in RICs-MAF and the enrichment of endogenous ribosomal status. These observations suggested that RICs-MAF might have experienced a non-canonical multipotent state during lineage conversion. In sum, we report a unique approach of an exo-ribosome-mediated plastic state of MAF that is amenable to multi-lineage conversion.

## 1. Introduction

Cellular plasticity refers to the cellular potential of a cell to transform from one phenotype to another in response to external stimuli [1]. The extent of cell plasticity and changes in cellular fate contribute to crucial biological phenomena in mammals. Historically, it was widely believed that cell differentiation is irreversible. This belief was revised by George Adami, who proposed that fully differentiated cells might re-enter the cell cycle and acquire the regenerative capacity to alter their cell fate under stress condition [1]. Since then, several studies have been carried out to investigate the plasticity of somatic cells and their acquisition of stem cell-like characteristics in different contexts of cellular development and pathological conditions [2,3].

Fibroblasts are ubiquitous cells, found in almost all types of tissues and identified by their characteristic elongated spindle-shaped morphology. These cells can be isolated from various tissues, which have roughly identical morphology and functionality [4]. They possess heterogenous populations and widely diverse genetic profiles which act crucially in development [5]. Fibroblasts were considered terminally differentiated cells until the last decade. Several reports have been published on reversing the fate of fibroblasts by different approaches, which demonstrates that fibroblasts can be differentiated into adipogenic, osteogenic, and chondrogenic lineages [6,7,8]. Approaches, such as incorporating transcription factors, i.e., Oct4, Sox2, Klf4, c-Myc (OSKM), or lineage-specific factors and introducing small molecules, have been covered in numerous former reports [9]. However, all of these have plausible limitations and room for improvement [10].

Turning to the “Ribosome”, which is commonly known as the translational machine, has recently gained attention for its other functions. Several reports have stated that individual ribosomal proteins can be involved in a variety of molecular processes in addition to translation [11,12]. Apart from these, a unique role of ribosomes in multipotency induction was reported by our lab in 2018. Here, the exogenous ribosome has been introduced into trypsinized mammalian fibroblasts, i.e., human dermal fibroblasts (HDF), mouse embryonic fibroblasts (MEF), and expressed stemness markers like Oct4, Nanog, and Sox2, followed by differentiation into three germ-layer-derived cells [13]. Ribosomes, regardless of their source, have been observed to induce such stem cell-like features [13]. However, the universality of multipotency induction and lineage conversion by ribosome incorporation in other somatic cells is yet to be explored. Aiming to explore this, mouse adult fibroblasts (MAF) were isolated from ear pinnae and studied here for lineage conversion by ribosome incorporation. Unlike MEF or tail-tip fibroblasts, MAF is infrequently reported. Recently, MAF have been deployed as preferrable alternatives in a few reports for functions like cellular senescence, wound healing, regenerative drug discovery, etc. [14,15,16]. In these reports, MAF served as true biological replicates of adult model organisms compared with standard cell lines like embryonic fibroblasts. Fibroblasts have been known for cellular heterogeneity and the tissue-specific properties of fibroblasts may change with mouse age [14]. Considering these, we hypothesized that ribosome incorporation to MAF could result in different trajectories of lineage conversion compared to our previous works.

This report sheds new light on describing in vitro cell generation through ribosome-mediated multipotency. Firstly, we discussed the optimal conditions for ribosome-induced cell cluster formation. These ribosome-induced cell clusters (RICs) are distinct from pluripotent cells. Next, placed into a proper experimental setup, these clusters were differentiated into multi-lineage cells with reorganized cytoskeletal protein patterns. Remarkably, we observed the conversion of cell fate that experienced a rare multipotent stem cell-like state. This ribosome-induced plasticity of MAF is reported here to decipher a novel role of the ribosome in lineage conversion and to delineate the summarized process of cell generation using extrinsic materials.

## 2. Materials and Methods

### 2.1. Isolation of Mouse Adult Fibroblasts (MAF)

Mouse ears (~1 cm radius) or ear punches were collected from female mice aged 3–5 months. The protocol of fibroblast isolation was followed as previously reported with minor modifications [17]. Briefly, ears were washed with 70% ethanol and then air dried. Hairs were removed from the ears by using scissors. Next, ears were washed with complete medium prepared as Dulbecco’s Modified Eagle Medium/DMEM (Gibco, Emeryville, CA, USA) with 10% Fetal Calf Serum/FCS and 0.1% Gentamicin (Sigma-Aldrich, St. Louis, MO, USA). Using scissors, ears were cut into pieces ~3 mm or smaller in size. These finely chopped earpieces were transferred to 2 mL tubes and then incubated into enzyme solution of 2.5 mg/mL Collagenase A (Roche Diagnostics, Mannheim, Germany), 20 mg/mL Pronase (Roche Diagnostics, Mannheim, Germany) and dissolved in complete medium for 90 minutes (min) at 200 revolutions per minute (rpm) in horizontal shaker at 37 °C. Cell suspension was prepared from digested ear tissues by using a 70 μm cell strainer and 2.5 mL syringe plunger. Cells were centrifuged at 800× *g*, 5 min, room temperature (RT), two times in the complete medium. Finally, cells were resuspended in 10 mL of complete medium with 10 μL Amphotericin B (stock solution: 250 μg/mL, Sigma) and incubated at 37 °C in a humidified 5% CO_2_ incubator for 48–72 h (h). The dishes were checked for cell attachment and the medium was replaced after 72 h. The cells were sub-cultured (after 72–96 h of inoculation) until the culture reached nearly 80% confluency. Next, the cells could be passaged or stocked as per the experimental design (the steps are illustrated in Supplementary Figure S1). The guidelines of the Committee on Animal Research at Kyushu University, Japan, were followed for performing cell culture experiments. All experiments were performed with independently prepared biological replicates of MAF cells (n = 3 for each condition). In addition, triplicate sets (technical replicates) were tested for each experiment.

### 2.2. Isolation and Purification of Ribosomes from Bacterial Culture

Tetra-(His)6-tagged ribosomes were isolated from *Escherichia coli (E. coli*) JE28 bacterial strain, which was a kind gift from the Department of Cell and Molecular Biology, Uppsala University, Sweden. The detailed protocol of isolation and purification of ribosomes from *E. coli* JE28 was previously described [13,18]. For affinity purification, a complete His-tag purification column (Roche Diagnostics, Mannheim, Germany) was used.

### 2.3. Formation of RICs-MAF (Ribosome-Incorporated Cell Clusters of Mouse Adult Fibroblasts)

MAF were maintained in DMEM complete medium, and cells at passage 5 were used for experiments. Each time ~90% confluent cultures were trypsinized and 1.0 × 10^5^ cells were inoculated in 500 μL of PluriSTEM^TM^ Human ES/iPS cell medium (Cat. SCM130, Merck, Darmstadt, Germany) per well of a 4-well (2 cm) culture dish (Thermo Scientific Nunc^TM^ Cell Culture Dishes, New York, NY, USA) after mixing with an optimized amount of purified His-tagged ribosomes. After 48 h of ribosome incorporation, 200 μL of fresh PluriSTEM^TM^ Human ES/iPS cell medium was added to each well. After 72 h of ribosome incorporation, the culture medium was replaced (half of its amount per well) in every 48–72 h interval. All immunostaining, protein expression assay, in vitro differentiation assay, real time-PCR (RT-PCR), quantitative real-time PCR (qRT-PCR), and RNA-sequencing were performed using the Control-MAF cells and RICs-MAF cultured in PluriSTEM^TM^ Human ES/iPS cell medium for 14 days (D). Control-MAF and RICs-MAF were maintained in uniform experimental conditions for replicated sets. Live imaging of ribosome-induced cell cluster formation was performed with a Keyence BZ-X800E microscope (4X lens) and the time lapse video was analyzed by BZ-X800E analysis software. The videos were taken at 6 frames (10 min) per hour continued for 24 h.

### 2.4. Identification of Ribosome Incorporation into RICs-MAF

The cell clusters induced by His-tag ribosomes were tested for ribosome incorporation. After 14 days of maintenance, RICs-MAF were fixed with 4% Paraformaldehyde (PFA) for 15 min at RT, washed with 1X Phosphate-Buffered Saline (PBS), and these clusters were incubated in 20% sucrose overnight (O/N) at 4 °C, on a shaker. Later, these clusters were set to cryoblocks using Tissue-Tek O.C.T. compound in cryomolds (Sakura Finetek USA, Inc., Torrance, CA, USA), and 10 μm sections were prepared. For immunocytochemistry, sections were treated with 5% skim milk in 1X PBS-0.1% Triton X-100 for 1 h at RT. The samples were incubated with primary antibody anti-6× His (Abcam, Cambridge, UK) at dilution 1: 200 O/N at 4 °C. After incubation, the samples were washed three times with 1X PBS-0.1% Triton X-100. Then, they were incubated with respective secondary antibodies at dilution 1: 800 for 2 h, at RT, in dark conditions. After completing the incubation period, samples were washed three times with 1X PBS-0.1% Triton X-100. Next, Hoechst 33342 (Life Technologies, San Francisco, CA, USA) was set at dilution 1: 1000 in 1X PBS for 15 min at RT, in dark conditions to incubate the samples. For confocal microscopy, a Leica TCS SP8 STED (Leica Biosystem, Wetzlar, Germany) confocal super-resolution microscope was used. Images were postprocessed by Leica LAS X software. All images were taken with the lowest pixel saturation. Staining and imaging conditions were similar during the study.

### 2.5. Alkaline Phosphatase Staining (AP Staining)

AP staining was performed as described in the manual of the AP staining kit (Systems Biosciences, Palo Alto, CA, USA). In brief, RICs-MAF at D3 and D14 from individual sets were fixed with fixation solution (supplied with kit) for 10 min at RT, washed twice with 1X PBS and incubated with AP staining solution for 30 min, at RT under dark conditions. After incubation, clusters were washed twice with 1X PBS and images were captured by Leica microscope (DM IL LED Fluorescence Inverted Laboratory Microscope).

### 2.6. In Vitro Differentiation of RICs-MAF to Mesodermal Lineage Cells

To test lineage-specific differentiation, RICs-MAF were maintained in PluriSTEM^TM^ Human ES/iPS cell medium for 14 days before being set to the differentiation assay. In each well of a 24-well dish (Corning, Durham, NC, USA), 50–60 clusters were transferred in the specific differentiation induction medium while control cells were also suspended in a similar ratio. For in vitro differentiation, Adipocyte Differentiation Basal Medium StemPro^®^ (Gibco, Cat. A10410-01), Osteoblast Differentiation Basal Medium StemPro^®^ (Gibco, Cat. A10069-01) and Chondrocyte Differentiation Basal Medium StemPro^®^ (Gibco, Cat. A10069-01) were used to differentiate RICs-MAF into adipocytes, osteoblasts, and chondrocytes, respectively. The culture medium was replaced (half of its amount per well) at every 48–72 h interval. RICs-MAF were cultured for 14 days to differentiate into adipocytes and then stained with Oil Red O (Sigma-Aldrich, Lot. SLBP5248V). To differentiate into osteoblasts and chondrocytes, RICs-MAF were maintained for 21 days. Then, osteoblasts were stained with Alizarin Red S (Sigma-Aldrich, Lot. A5533-25G) and chondrocytes were stained with Alcian Blue Stain Solution (Nacalai Tesque, San Diego, CA, USA, Lot. L1E7365). Also, another set of differentiated samples were prepared for RNA isolation and cDNA preparation, maintaining similar conditions. Replicates of each set were used for immunostaining as well.

### 2.7. In Vitro Differentiation of RICs-MAF to Ectodermal Lineage (Neural) Cells

For cross-lineage differentiation, RICs-MAF were maintained, and at least 10 clusters were transferred per well of the 96-well dish (Greiner Cell Culture Microplate, 96-well, F-bottom, Stuttgart, Germany) as described in the section above. The neural induction media were prepared in DMEM medium containing 0.5% FCS, supplemented with 20 ng/mL bFGF (basic fibroblast growth factor, Sigma) singly or in combination with 10 ng/mL EGF (epidermal growth factor, Sigma). To differentiate into neural cells, RICs-MAF were maintained for 21 days while the medium was replaced (half of its amount per well) every 48–72 h interval. Later, these cells were checked by immunostaining as mentioned later.

### 2.8. Real Time-PCR and Quantitative Real Time-PCR

Total RNA of Control-MAF and RICs-MAF were purified at D14 and D21 (after differentiation) using the RNeasy Plus Mini Kit (Qiagen, Germantown, MD, USA), following the manufacturer’s protocol. Next, each cDNA was synthesized using 500 ng RNA, Oligo (dt) primers (Life Technologies), dNTP (deoxynucleotide triphosphate, Invitrogen, Carlsbad, CA, USA), 5× First Strand buffer (Invitrogen) and 0.1 M DTT (dithiothreitol, Invitrogen) and SuperScript II Rnase-H-Reverse Transcriptase (Thermo Fisher). These cDNAs were used for RT-PCR and qRT-PCR. The conditions set for RT-PCR were 98 °C for 2 min, 55 °C for 30 s, 72 °C for 1.5 min, and 72 °C for 3 min, involving 35 cycles of amplification. For qRT-PCR, Luna Universal qPCR One Step RT-qPCR Kit (New England Biolabs, Ipswich, MA, USA) was used. Here, 40 cycles of amplification (95 °C for 15 s, 57 °C for 60 s) were performed on a Step One Real-Time PCR System and analyzed by Step One Software version 2.3 (Thermo Fisher Scientific). Gene expression levels were normalized to those of Gapdh. Each sample was analyzed in triplicate (biological replicates). The comparative Ct Method (2^−ΔΔCt^) was applied, where ΔΔCt = Δ Ct (sample control) − Δ Ct (housekeeping control, Gapdh). Non-parametric unpaired Student’s *t*-test was employed to compare expressions (n = 3; * *p* < 0.05, ** *p* < 0.005, *** *p* < 0.0005). The graphs of qRT-PCR-analysis were prepared in GraphPad Prism version 10.2.3. The primers are listed in Supplementary Table S1.

### 2.9. Immunocytochemistry

For immunocytochemistry, RICs-MAF cluster sections (10 μm) were treated with 5% skim milk in 1X PBS-0.1% Triton X-100 for 1 h, RT. The samples were incubated with individual primary antibody for O/N at 4 °C (Supplementary Table S2). After three washes with 1X PBS-0.1% Triton X-100, the sections were incubated for 2 h at RT, in dark conditions with respective secondary antibody (Supplementary Table S2).

To perform immunocytochemistry of in vitro differentiated RICs-MAF, each category of cells was fixed with 4% PFA for 15 min at RT, washed with 1X PBS and treated with 5% skim milk in 1X PBS-0.1% Triton X-100 for 1 h, RT. These cells were incubated with individual primary antibody (Supplementary Table S2) O/N at 4 °C. Next, samples were washed with 1X PBS-0.1% Triton X-100 three times. Secondary antibody was used as per suitability. For adipocytes, Lipi-Green (Dojindo Laboratories, Kumamoto, Japan) was added instead of primary antibody (1: 100) and incubated for 30 min at RT in dark conditions, while the initial steps were followed as mentioned above. Afterwards, samples were incubated with Hoechst 33342 (1: 1000) for 15 min at RT in dark conditions. These samples were washed with 1X PBS. Finally, mounting solution was used to prepare the slides for imaging.

### 2.10. Western Blot

Control-MAF and RICs-MAF (1.0 × 10^5^ cells/500 μL, 50 μg of ribosome, n = 3) were maintained in PluriSTEM^TM^ Human ES/iPS cell medium (D14). At the time of sample collection, control cells and RICs were washed with 1X PBS (two times). Next, samples were collected in 500 μL IP Lysis Buffer (for 1× stock, 150 mM NaCl, 20 mM Tris-HCl, pH 7.5, 1.5 mM CaCl_2,_ 1.5 mM MgCl_2_, 0.1% Triton X-100, 0.1% CHAPS, 5% glycerol and 0.1% BSA) and lysed by vigorous pipetting on ice for 2–3 min. Protein concentration was measured by Bradford assay (Bio-Rad, Hercules, CA, USA) at OD 595 nm. Loading sample was prepared by 2X SDS sample loading buffer (4% *w*/*v* sodium dodecyl sulfate, 20% (*v*/*v*) glycerol, 0.2% (*w*/*v*) bromophenol blue, 100 mM Tris-HCl, pH 6.8, 200 mM β-mercaptoethanol) with 2 μg protein for each sample, then heated at 95 °C for 10 min. Samples were separated via 12% gel and transferred into a PVDF (polyvinylidene difluoride) membrane. The transferred PVDF membrane was blocked with 5% skim milk in 1X PBS-0.1% Tween-20 (PBS-T) for 1 h at RT with gentle shaking on a horizontal shaker. Later, membranes were incubated at 4 °C O/N in a horizontal shaker with individual primary antibody (Supplementary Table S3). Each membrane was washed with a generous volume of PBS-T three times and incubated with respective secondary antibody for 2 h at RT in a horizontal shaker (Supplementary Table S3). Afterwards, each membrane was washed with PBS-T three times and the band was detected by ImmunoStar (Fujifilm, Tokyo, Japan). Western blot images were analyzed by ImageJ (bundled with Java8).

### 2.11. RNA-Sequencing (RNA-Seq)

Control-MAF and RICs-MAF were prepared for RNA-Seq as per the RNA-Seq sample preparation guidelines from Active Motif, Carlsbad, CA, USA. In brief, Control-MAF (1.0 × 10^5^ cells/500 μL) and RICs-MAF (1.0 × 10^5^ cells/500 μL, 50 μg of ribosome) were maintained in PluriSTEM^TM^ Human ES/iPS cell medium for 14 days. Replicates were prepared to reach cell number 2 × 10^6^ cells. Control-MAF cells were very gently scraped from the culture dish while RICs-MAF were collected in their floating state. Both samples were centrifuged at 800× *g*, 4 °C for 5 min to decant residual culture media. Samples were resuspended in chilled 1X PBS and spun down similarly. Afterwards, cell pellets were frozen in liquid nitrogen and stored at −80 °C until RNA isolation. The RNA quality was strictly maintained having a RIN score (RNA Integrity Number) greater than 8.0 for library preparation.

Next, RNA libraries were prepared from the purified RNA using the TruSeq Stranded mRNA sample preparation kit (Illumina). RNA-sequencing (150 nt paired-end reads) was performed utilizing a NovaSeq 6000 System (Illumina), following the guidelines provided by the manufacturer. FastQ reads generated from the samples were subjected to trimming to eliminate low-quality regions (defined by a Phred quality score cut off, Q = 20) and adaptor sequences using the FastP tool (0.23.2) [19]. Subsequently, the trimmed paired-end reads for each sample were aligned to the GRCm39 reference genome using the HISAT2 program (version 2.2.1) [20]. Transcript quantification was performed by Limma-Voom Package [21]. *p* < 0.01 and FDR < 0.1 cut-off values were used for the significant gene selection for downstream analysis. Overrepresentation-based gene enrichment analysis was performed for differentially expressed genes (DEG) by the Webgestalt tool [22].

## 3. Results

### 3.1. Incorporation of Ribosome into MAF

**Figure 1:**
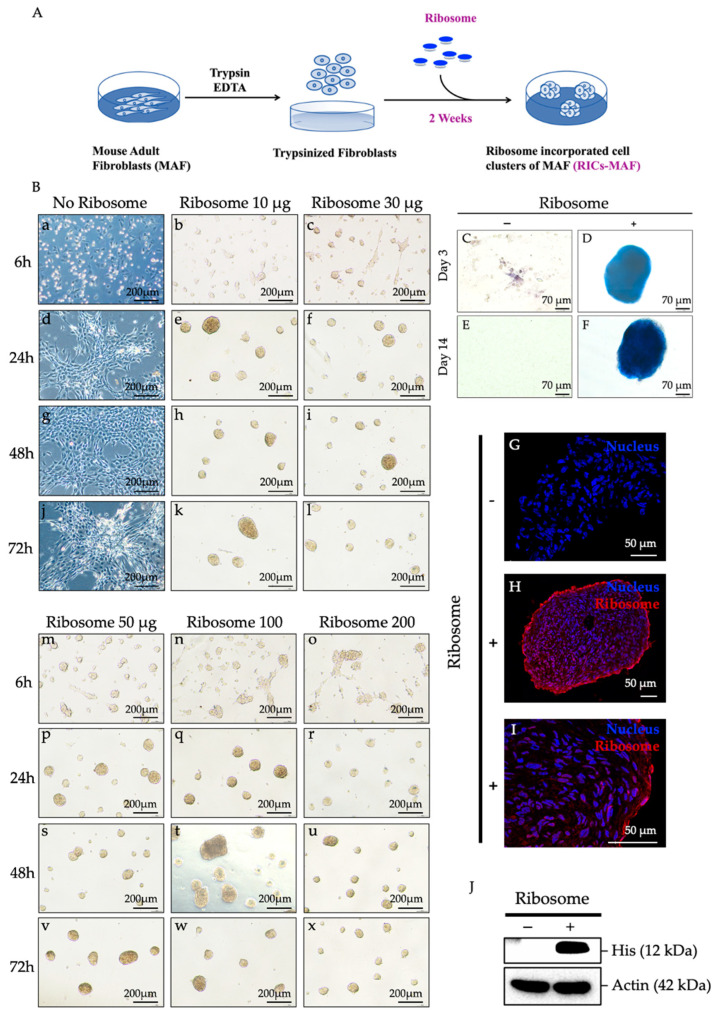
Ribosome incorporation forms clusters in mouse adult fibroblasts. (**A**) Illustration of ribosome incorporation methodology. (**B**) Optimization of cluster formation based on ribosome amount and time; ribosome ranging from 10 µg to 200 µg was added into 1.0 × 10^5^ cell/per well of a 4-well dish. Cluster formation was recorded at 6 h, 24 h, 48 h, and 72 h after incorporation. (**C**–**F**) AP staining of RICs-MAF showed positive staining while Control-MAF did not show any staining. (**G**–**I**) Exo-ribosome expression in RICs-MAF, immunocytochemistry, and western blot were performed using anti-6x His antibody that recognizes His-tagged-L-12 ribosomal protein that confirmed the incorporation of ribosome into MAF at D14. (**G**) Control-MAF (negative control) and (**H**,**I**) intracellular localization of ribosome was observed in the cytoplasm and the nucleus, respectively. (**J**) Western blot also confirmed ribosome incorporation in MAF while Control-MAF did not show any band.

Previously, we reported that HDF, MEF, glioma cells, and cancer cells (such as A549, H-111-TC, MCF7) formed cell clusters by ribosome incorporation. In line with former works, the effects of ribosome incorporation on MAF were examined here. The cells were trypsinized and mixed with ribosomes on culture plates (Figure 1A) to check their cluster formation ability. First, the amount of ribosomes and the time required to form cell clusters were optimized. Ribosomes were incorporated as 10 μg, 30 μg, 50 μg, 100 μg, and 200 μg for 1.0 × 10^5^ cells (per well of 4-well dish/500 μL media), and time was observed as 6 h, 24 h, 48 h, and 72 h. Cell clusters were observed to form within 6 h after ribosome incorporation with a minimum amount of 10 μg. A definite morphological alteration was observed between Control-MAF and RICs-MAF after ribosome incorporation (Supplementary Video S12 for Control-MAF and Supplementary Video S13 for RICs-MAF, 24 h time-lapse). Cluster formation was observed within 6 h (Figure 1B(a–c,m–o)), and observation continued to 24 h (Figure 1B(d–f,p–r)), 48 h (Figure 1B(g–i,s–u)), and 72 h (Figure 1B(j–l,v–x)). The clusters were estimated to be approximately 100–200 μm in diameter, and RICs-MAF remained in their floating cluster form until they were transferred to the differentiation medium or collected for analysis until D14.

Meanwhile, Control-MAF did not make any cluster, remained attached to the dish, and proliferated uninterruptedly (72 h and D14). Our results suggested 50 µg ribosome as the optimum requirement of ribosome to induce morphologically healthy cluster formation. For the rest of this study, 50 μg of ribosome was incorporated for 1.0 × 10^5^ cells (per well of 4-well dish/500 μL media), maintained from D3 to D14, at which point samples were collected for analysis. These cell clusters were tested by AP staining at D3 (Figure 1C,D) and D14 (Figure 1E,F). RICs-MAF showed strong positive staining (Figure 1D,F) while Control-MAF did not show any staining (Figure 1C,E). Next, immunocytochemistry was performed using anti-6× His antibody that recognizes His-tagged-L-12 ribosomal protein to confirm the incorporation of ribosome into MAF cells at D14 (Figure 1G–I, Supplementary Figure S11). We observed when exogenous ribosome was incorporated into a cell, this ribosome could reside in the nucleus (Figure 1I) and the cytoplasm simultaneously (Figure 1H, Supplementary Video S14 10 µm section-Z-stack imaging of RICs-MAF, Supplementary Video S15 zoomed central region-Z-stack imaging of RICs-MAF). No staining was observed in Control-MAF (Figure 1G). Western blot also confirmed ribosome incorporation in MAF (His-tagged expression) while Control-MAF did not show any band (Figure 1J).

We would like to mention the ribosome used in this study has conserved structural (whole ribosome) and functional activity. Though we could detect its existence in the nucleus and the cytoplasm of fibroblast cell by His-tag expression, the consequences of this incorporated exogeneous ribosome during plasticity induction remain speculative.

### 3.2. RICs-MAF Differentiated into Multi-Lineage Cells

Following the cluster formation from adult fibroblasts, we investigated whether RICs-MAF could be differentiated into other cell lineages. Previously, HDF were observed to differentiate into three germ-layer-derived cells after ribosome incorporation [13]. For each set, at least 50 clusters were randomly selected and transferred to designated differentiation induction media (per well of a 24-well dish). Clusters were cultured from D14 (adipocytes) to D21 (osteoblasts and chondrocytes). Afterward, these sets were stained with lineage-specific dyes to observe differentiated cells.

**Figure 2:**
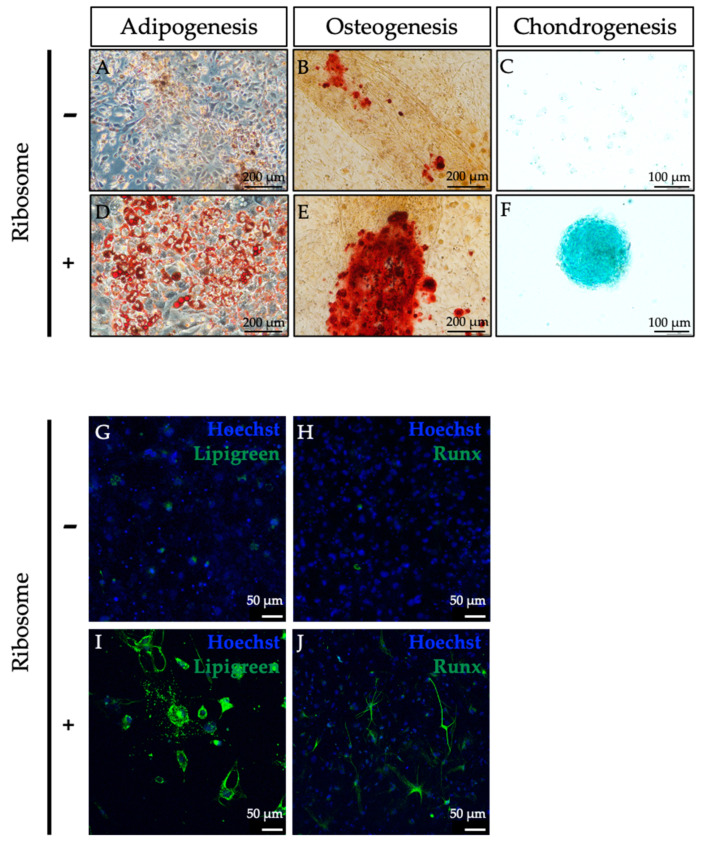
Ribosome-incorporated mouse adult fibroblasts differentiated to adipocytes, osteoblasts, and chondrocytes. (**A**–**F**) For each set, 50 clusters were transferred to a specific differentiation induction medium (per well of 24-well dish) and they were left to differentiate from D14 (adipocytes) to D21 (osteoblasts and chondrocytes). Differentiated adipocytes were stained by Oil Red O staining (**A**,**D**). For osteoblasts, Alizarin Red staining (**B**,**E**) was performed, and for chondrocytes, Alcian Blue staining (**C**,**F**) was performed. Optical light microscopic images are shown (original magnification: ×200, ×100, scale bar: 200 µm, 100 µm). (**G**–**J**) Immunostaining of adipocytes with Lipi-Green (**G**,**I**) and osteoblasts with Runx antibody (**H**,**J**) were observed in respective RICs-MAF differentiated cells. Hoechst 33342 was used for nuclei counterstaining. Scale bar: 50 µm.

For all types, we observed the clusters were attached to the dish, leaving their floating state, and gradually differentiated to designated lineage cells. For adipocytes, RICs-MAF clusters initially attached and remained in shape. Consecutively the peripheral regions of clusters were dissociated in many clusters. Then, the formation of lipid droplets was observed from these cluster-dissociated cells. These differentiated cells were stained by Oil Red O staining and compared to the Control-MAF (Figure 2A,D). For osteoblasts, RICs-MAF clusters were attached, and the peripheral regions of clusters were dissociated. These differentiated cells made thread-like structures with adjacent clusters and deposited a whitish calcified bone matrix. During Alizarin Red staining, all differentiated cells were stained strongly while some cells of Control-MAF stained faintly (Figure 2B,E). Likewise, for chondrocytes, RICs-MAF clusters were attached, but the peripheral regions of clusters were not dissociated.

The cell clusters maintained their shape and showed positive staining in Alcian Blue staining while control cells showed almost negative staining (Figure 2C,F).

Later, immunostaining of RICs-MAF differentiated adipocytes was performed with Lipi-Green (Figure 2G,I) and osteoblasts were performed to test Runx (osteoblast marker, runt-related transcription factor) expression (Figure 2H,J). In both cases, these differentiated cells showed convincing expressions. Subsequently, RT-PCR data revealed an altered expression of the fibroblast marker gene (fibroblast surface protein, FAP) in RICs-MAF compared to Control-MAF (D14) (Supplementary Figure S2). In addition, lineage-specific marker gene expression was tested after differentiation (D21). Differentiated cells generated from RICs-MAF expressed Adiponectin (adipogenesis marker), Osteocalcin (osteogenesis marker), and Col10a1 (chondrogenesis marker), while Control-MAF cells retained their fibroblast characteristics (Supplementary Figure S2). These observations suggested considerable lineage conversion of RICs-MAF differentiated cells from their fibroblast origin.

### 3.3. RICs-MAF Differentiated into Neural Cells

**Figure 3:**
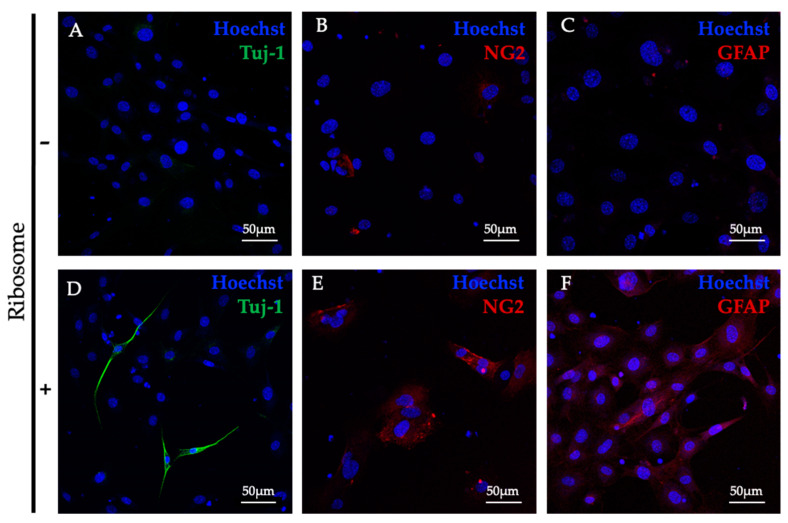
Neural differentiation of ribosome-incorporated mouse adult fibroblasts. Control-MAF showed slightly spontaneous differentiation for neurons (**A**), oligodendrocytes (**B**), and astrocytes (**C**), while RICs-MAF differentiated into neurons marked by Tuj1 (**D**), oligodendrocytes marked by NG2 (**E**)**,** and astrocytes marked by GFAP (**F**), respectively, cultured in neural differentiation induction medium for D21. Hoechst 33342 was used for nuclei counterstaining. Scale bar: 50 µm.

Previously, ribosome-incorporated HDF were observed to differentiate into ectodermal neurons [13]. Additionally, an earlier report observed the direct conversion of mouse tail-tip fibroblasts to functional neurons by defined factors [23]. At this stage, we tested whether bacterial ribosome incorporation could differentiate MAF to cross-lineage cells. For this, RICs-MAF (D14) were randomly selected (at least 10 clusters) and transferred to a designated neural differentiation induction medium (per well of 96-well dish/200 μL media). The clusters were maintained for D21. Similarly to other differentiated cell lineages, RICs-MAF clusters were attached to the dish leaving their floating state. Then, RICs-MAF clusters were dissociated slowly from their peripheral edges and altered morphologically. To examine whether these differentiated cells expressed neural cell-specific markers, a few common neural cell markers were tested for immunostaining (Figure 3A–F). We observed these RICs-MAF differentiated cells were positive for Tuj1 (neurons) (Figure 3D), NG2 (oligodendrocytes) (Figure 3E), and GFAP (astrocytes) (Figure 3F). However, their Control-MAF counterparts (Figure 3A–C, respectively) were also observed to be slightly differentiated and expressed neural cell markers.

Overall, RICs-MAF could be differentiated into mesodermal lineage cells, i.e., adipocytes, osteoblasts, chondrocytes, and cross-lineage ectodermal cells, i.e., neural cells. These observations suggested ribosomes can induce lineage-convertible, phylogenetic-relationship-independent cells from MAF.

### 3.4. RICs-MAF Attenuated Fibroblast Characteristics

**Figure 4:**
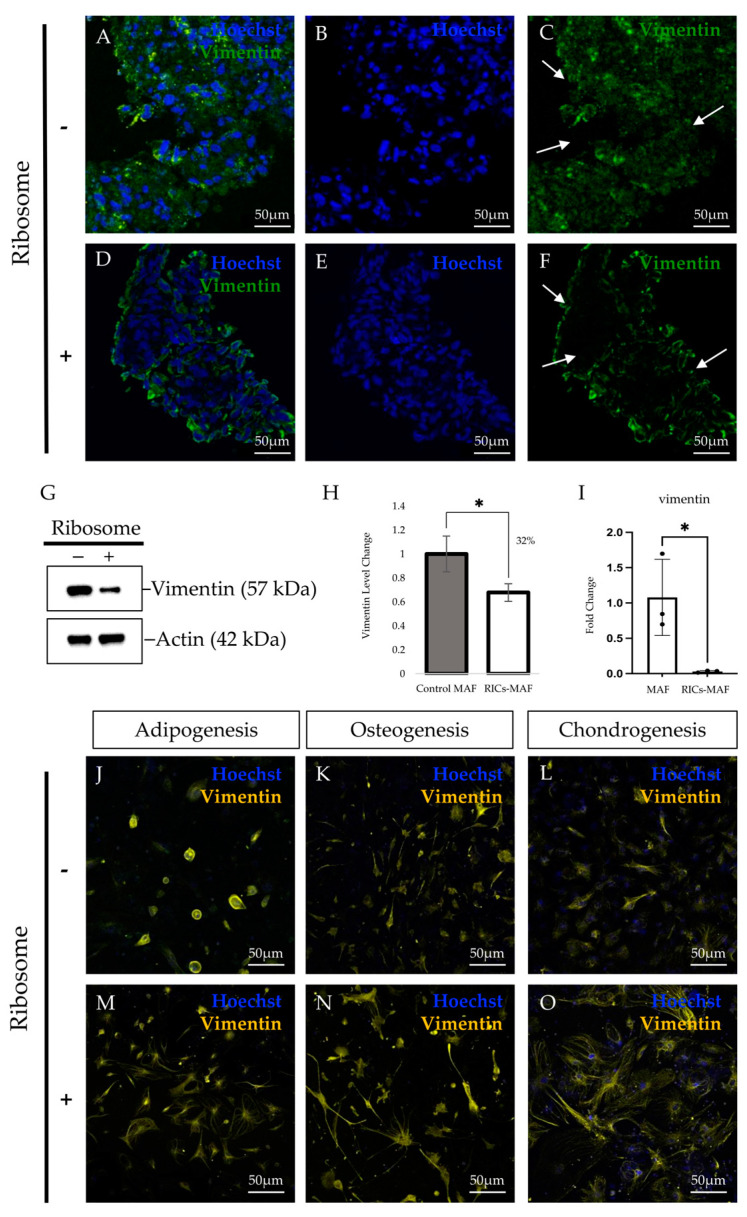
Ribosome-incorporated mouse adult fibroblasts diminished fibroblast characteristics. (**A**–**C**) Control-MAF and (**D**–**F**) RICs-MAF (D14) were stained with anti-vimentin antibody. Considerable alteration of vimentin expression was observed in RICs-MAF than Control-MAF. Control-MAF showed a high level of vimentin while altered expression was observed in RICs-MAF through immunostaining. Arrowheads indicate the reduction of vimentin in RICs-MAF. (**G**,**H**) This observation was further examined by western blotting. Western blot of total cellular protein lysates of Control-MAF and RICs-MAF revealed significantly reduced (~32%) vimentin expression (**H**). (**I**) qRT-PCR analysis also showed a significant reduction in vimentin expression. The unpaired non-parametric Student’s *t*-test was employed to compare expression (n = 3; * *p* < 0.05). Therefore, fibroblast characteristics were noticed to be abolished in RICs-MAF (**H**,**I**). (**J**–**O**) Next, differentiated cells of RICs-MAF to adipocytes (**J**,**M**), osteoblasts (**K**,**N**), and chondrocytes (**L**,**O**) were stained with anti-vimentin antibody. Remodeled pattern of vimentin expression was observed in each differentiated cell type from RICs-MAF than their Control-MAF counterparts. Fluorescence microscopic images are shown (scale bar: 50 µm).

Aiming to know more about RICs-MAF features and their differentiation ability, we next examined fibroblast characteristics in RICs-MAF after 14 days of ribosome incorporation. The cell clusters were collected in their floating state, and samples were prepared to check fibroblast marker expression by immunostaining and western blot. By immunostaining, we observed the expression of fibroblast markers; however, vimentin was different between Control-MAF (Figure 4A–C) and RICs-MAF (Figure 4D–F). Western blot data showed a reduced level of vimentin in RICs-MAF (~32%) (Figure 4G,H). Later, RT-PCR and qRT-PCR analysis for vimentin expression also supported this observation (Supplementary Figure S3 and Figure 4I).

Subsequently, we observed distinct patterns of vimentin expression in RICs-MAF differentiated adipocytes (Figure 4J,M), osteoblasts (Figure 4K,N), and chondrocytes (Figure 4L,O). Moreover, the cytoskeletal protein marker, actin, was also altered in RICs-MAF differentiated adipocytes (Supplementary Figure S4A,D), osteoblasts (Supplementary Figure S4B,E), and chondrocytes (Supplementary Figure S4C,F). Previous studies have shown extensive cytoskeletal networks and spread morphology of actin and vimentin expression in fibroblasts [24]. Adipocytes, osteocytes, and chondrocytes also possess distinct spatial distribution and expression of key cytoskeletal proteins, which serve as markers for each differentiated phenotype [25,26,27]. Based on the rare protein patterning observed here, we theorized that RICs-MAF differentiated cells might have experienced de novo remodeling of cytoskeletal proteins during lineage conversion.

Earlier, RT-PCR data also showed reduced expression of fibroblast surface protein marker (FAP) in RICs-MAF compared with Control-MAF (Supplementary Figure S2). Combined with prior data, it is likely that the reduction of the fibroblast phenotype was induced by ribosome. The faded fibroblast phenotype welcomed differentiation into multilineage cells, while incorporated ribosome maintained cellular differentiation ability in RICs-MAF clusters. In this context, our data suggested vimentin might have acquired a pro-stemness role in RICs-MAF to define lineage fate [28,29].

### 3.5. RICs-MAF Expressed Early Senescence Markers

**Figure 5:**
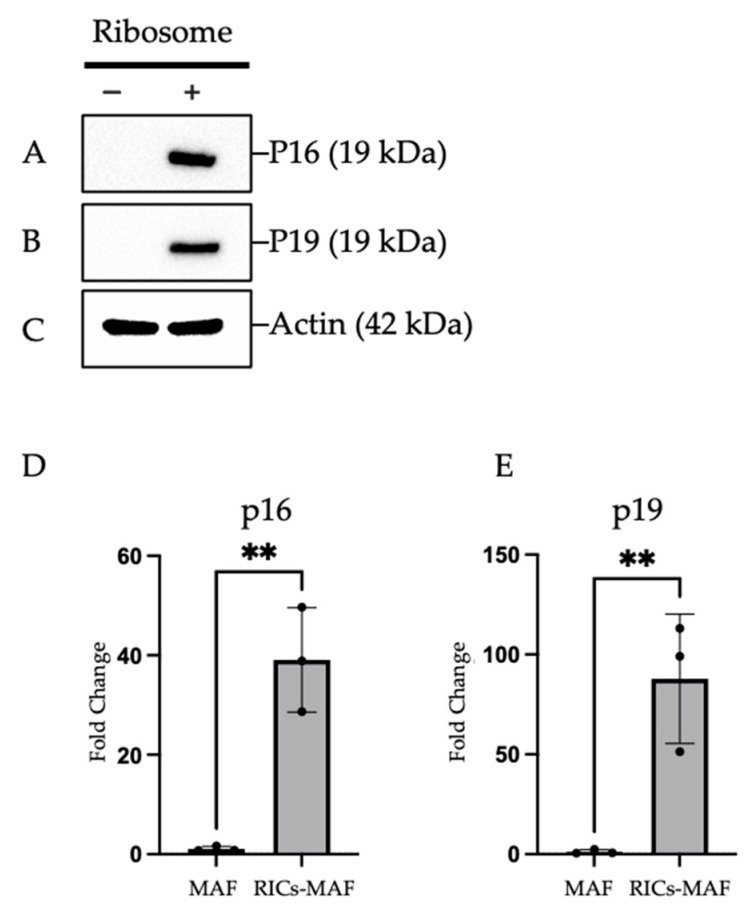
Ribosome-incorporated mouse adult fibroblasts expressed early senescence markers. (**A**–**C**) Total cellular protein lysates of Control-MAF and RICs-MAF (D14) were blotted with (**A**) anti-P16, (**B**) anti-P19, and (**C**) anti-Actin (housekeeping control) antibody. Expression of senescence markers P16 and P19 was observed in RICs-MAF but not in Control-MAF. (**D**,**E**) qRT-PCR analysis of p16 and p19 expression also showed significant upregulation of these genes in RICs-MAF. Unpaired non-parametric Student’s *t*-test was employed to compare expression (n = 3; ** *p* < 0.005). These results suggested that ribosome-incorporated MAF experienced suppressed cell proliferation, though these cells expressed early senescence markers to exhibit cell differentiation ability.

Our former studies observed the inability of RICs cells to proliferate, which was noticed by the simultaneous expression of renowned senescent markers [13]. Intrigued by this, we next tested the cellular status of RICs-MAF (D14). The cell clusters were collected in their floating state and samples were prepared to check the expression of senescence markers by western blot. We observed the expression of senescence markers P16 and P19 in RICs-MAF but not in Control-MAF (Figure 5A–C). Later, RT-PCR and qRT-PCR for p16 and p19 expression also supported this observation (Supplementary Figure S5 and Figure 5D,E). These results suggested ribosome-incorporated MAF cells experienced suppression of cell proliferation after cluster formation and remained stationary. They also expressed early senescence markers to exhibit cell differentiation ability.

### 3.6. RICs-MAF Acquired Rare Multipotent Stem Cell-like State during Lineage Conversion

**Figure 6:**
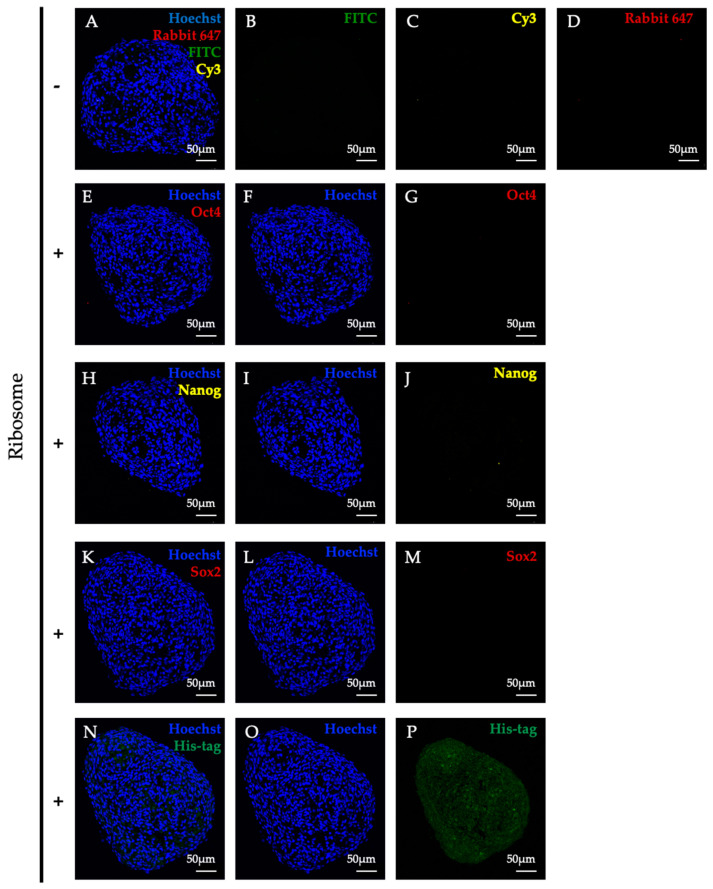
Typical multipotent state was not observed during lineage conversion in RICs-MAF (D14). RICs-MAF sections were stained with anti-Oct4, anti-Nanog, and anti-Sox2 antibody. His-tagged ribosome was detected with Anti-6x-His antibody, and nuclei staining was performed with Hoechst 33342. Respective secondary antibody control is presented here as top panel (**A**–**D**), expression of stemness markers, Oct4 (**E**–**G**), Nanog (**H**–**J**), and Sox2 (**K**–**M**) are presented sequentially. Neither of them were detected considerably. Ribosome incorporation is presented as the bottom panel (**N**–**P**). Fluorescence microscopic images are shown here (scale bar: 50 µm).

To gain insight into the course of alteration in cell characteristics following ribosome incorporation, we tested stemness marker expression in RICs-MAF. Previously, we have observed that bacterial ribosome incorporation induced renowned pluripotency markers (i.e., Oct4, Nanog, and Sox2) expression in HDF and MEF, which led to differentiation [13]. It was intriguing to explore whether similar induction occurred in MAF. Here, RICs-MAF were collected on D14, cryosectioned, and immunostained with antibodies (i.e., anti-Oct4, anti-Nanog, and anti-Sox2) individually. Surprisingly, our analysis revealed no noticeable expression of these markers in RICs-MAF. Compared with negative control (Figure 6A–D), the expression of Oct4 (Figure 6E–G), Nanog (Figure 6H–J), and Sox2 (Figure 6K–M) could not be detected by immunostaining in RICs-MAF, while ribosome incorporation was detected through His-tag expression (Figure 6N–P). We could detect stemness marker expression and confirm antibody specificity by testing ribosome-incorporated MEF (RICs-MEF) as a positive control (Supplementary Figure S6). In addition, Mouse IgG and Rabbit IgG were tested as negative controls along with second antibody controls (Supplementary Figure S7). At this point, we speculated there might be an alternative strategy for plasticity in RICs-MAF, and this prompted us to investigate the induction of other genes associated with multipotency in RICs-MAF.

**Figure 7:**
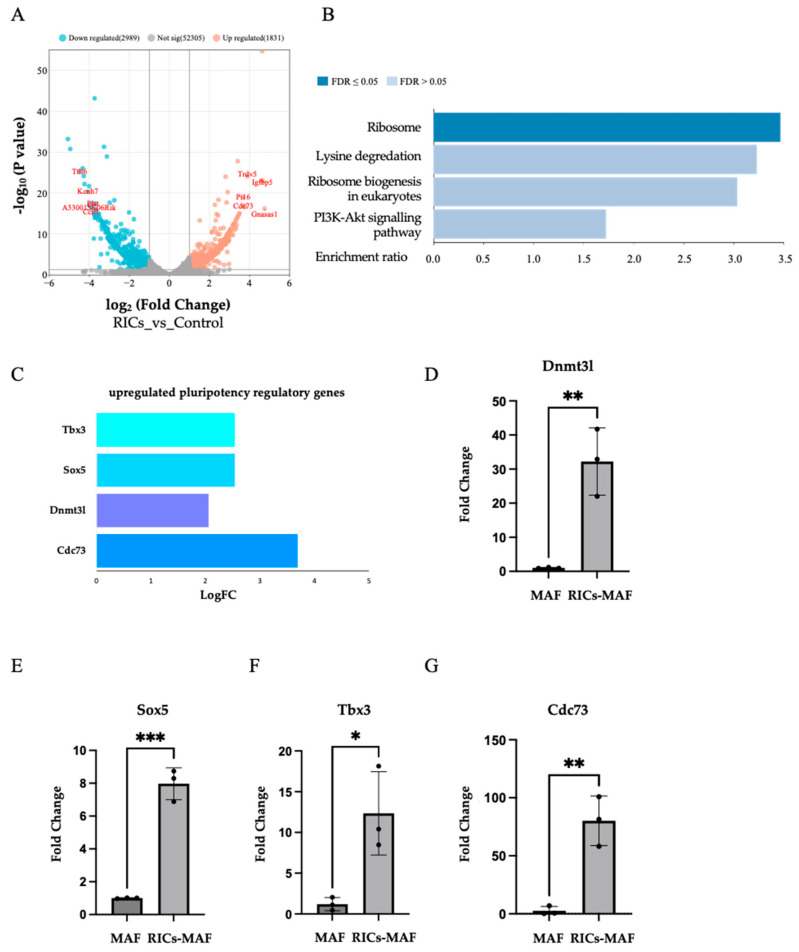
RNA-seq analysis revealed the stimulation of endogenous ribosomal gene and the acquisition of rare multipotent state in RICs-MAF. (**A**) RNA-sequencing and subsequent differential gene expression analysis. (**B**) Gene enrichment analysis of significantly upregulated genes in RICs-MAF under KEGG pathway categories, notably endogenous ribosome activity (FDR < 0.05) was observed. (**C**) Significant upregulation of stemness associated genes, including Dnmt3l, Sox5, Tbx3 and Cdc73 upon exposure to bacterial ribosome. These genes are known to play crucial roles in maintaining pluripotency and regulating cellular differentiation pathways. (**D**–**G**) qRT-PCR data also showed significant upregulation of Dnmt3l, Sox5, Tbx3 and Cdc73 showed similar but non-significant upregulation. Non-parametric unpaired Student’s *t*-test was employed to compare expressions (n = 3; * *p* < 0.05, ** *p* < 0.005, *** *p* < 0.0005). These observations suggested a potential mechanistic link between the induction of multipotency-associated genes and enriched ribosomal activity.

To elucidate this further, we performed RNA-sequencing and subsequent differential gene expression analysis (Figure 7A,B). Strikingly, our results demonstrated a significant upregulation of several stemness-associated genes, including Dnmt3l, Sox5, Tbx3, and Cdc73, upon exposure to bacterial ribosome (Figure 7C). Notably, these genes are known to play crucial roles in maintaining pluripotency and regulating cellular differentiation pathways [30,31,32,33]. Significant upregulation of Dnmt3l, Sox5, Tbx3, and Cdc73 was observed by qRT-PCR analysis (Figure 7D–G). Our RT-PCR data also showed similar observations (Supplementary Figure S8). Therefore, our data suggested bacterial ribosome influenced this rare multipotent state of MAF. In addition, our gene enrichment analysis of the significantly upregulated genes revealed a notable enrichment of the endogenous ribosome pathway (FDR < 0.05), which provided an insight for a potential mechanistic link between the induction of pluripotency-associated genes and ribosomal activity (Figure 7B).

Together, our findings suggest that exogenous ribosome stimulated MAF cell plasticity to differentiate into cells of different dermal lineages, while these cells acquired the expression of stemness-associated genes and enriched the endogenous ribosome pathway.

## 4. Discussion

Ribosome-mediated multipotency represents a game-changing approach in the field of cell fate reversal. The conventional approaches of cell fate change by transcription factors or small molecules include potential risks regarding genomic integration of oncogenic transcription factors, limited efficiency of vehicle-based gene delivery, context dependency, repetitive experiments to set optimal combinations of small molecules, expensive operational costs, strict ethical concerns, etc. [33,34,35]. Hence, the search for a novel way to induce cell fate conversion without incurring genetic modification has become the focus of intense research. In this perspective, ribosome-mediated multipotency merits a safer alternative approach to overcome these limitations.

This technique is simple, scalable, and accessible for research as well as clinical set up. Ribosomes are easy to purify from bacterial culture and can be delivered to trypsinized cells through endocytosis [13]. The non-invasive and non-integrating features of the technique have great potential for clinical application over viral-based delivery methods. This technique mimics the natural coexistence of mammals with bacteria and minimizes the risk of immune rejection [36]. This method offers versatility as it can be applied to cells from different sources [13,37,38,39]. Moreover, the utilization of bacterial substances can bypass ethical considerations on genomic modification and embryonic cell usage. This approach presents an ethically and socially perferrable alternative for regenerative medicine research.

Prior to this study, we have reported on the lineage conversion of HDF by lactic acid bacteria [36] and also discovered that “ribosome” acted as the inducing factor for such a phenomenon [13]. Later, we reported ribosome can induce multipotency in cancer cells and glioma cells [37,38,39]. However, it remained ambiguous whether cell fate reversal was affected by HDF or cancer cell phenotypes. Hence, this study was conducted to explore ribosome as a universal cellular stimulant for plasticity toward lineage independent cells from terminally differentiated cells. Here, we have focused on the feasibility of generating multilineage cells from native fibroblast cell; however, most studies regarding cell plasticity were performed in human cells or mouse embryonic cells, in which genetic (or epigenetic) and environmental differences might have an intervening role.

This study evaluated an occasionally studied cell, MAF, which is considered a terminally differentiated heterogenous cell. Its isolation from adult mouse ear pinnae has made it easier to collect samples without sacrificing the mouse. A small ear punch can be collected to prepare primary fibroblast culture and conduct experiments. Thus, we proposed its usage as an economically viable source for cell regeneration studies. Moreover, studying this cell enabled us to envisage the prospective therapeutic application of such ribosome-mediated multipotent cells in in vivo models.

Our former work showed the induction of stemness marker Oct4, Nanog, and Sox2 through ribosome incorporation, which altered cellular state during in vitro culture. Later, these cells differentiated into hepatic, muscle, and neural cells, respectively [13]. Although ribosome-mediated multipotency differs from conventional pluripotent stem cell features in some respects, further exploration of such ambiguous stemness was the aim of this study. Based on the present data, Oct4, Nanog, and Sox2 expression could not be detected in RICs-MAF by immunostaining and RNA-sequencing. However, we could detect the upregulation of Dnmt3l, Sox5, Tbx3, Cdc73, and several endogenous ribosomal proteins. The upregulation of genes such as Dnmt3l, Sox5, Tbx3, and Cdc73 have been reported to act on maintaining pluripotency and regulating cellular differentiation pathways in MEF, mouse dermal fibroblasts and mouse embryonic stem cells [30,31,32,40]. Dnmt3l is a member of the DNA methyltransferase family and is involved in establishing DNA methylation patterns during early embryonic development. Its upregulation may influence stemness by regulating epigenetic modifications that control gene expression patterns associated with pluripotency and differentiation [30]. Sox5 is a member of the Sox family of transcription factors, which play critical roles in embryonic development and cell fate determination. Sox5 has been associated with the regulation of stem cell pluripotency and differentiation. Its upregulation may contribute to the maintenance of stemness by regulating the expression of key pluripotency genes [31]. Tbx3 is known to be a transcription factor involved in embryonic development and has been implicated in the regulation of pluripotency. It may contribute to the maintenance of stemness by modulating the expression of genes involved in self-renewal and differentiation pathways [32,40]. Cdc73, also known as parafibromin, is a tumor suppressor gene that has been linked to the regulation of cell proliferation and differentiation. Its upregulation may influence stemness by modulating cell cycle progression and differentiation pathways [41]. We speculated that a rare harmony of these stemness-associated genes might be responsible for multipotency induction in this study. We also observed the absence of renowned proinflammatory signals in RICs-MAF which ruled out the possibility of inflammation-induced lineage conversion (Supplementary Table S7). By being insightful regarding these studies, recent observations led us to the paradigm of an alternative strategy that connects stemness acquisition and enriched ribosome status, through which endogenous bacterial ribosome influence the non-canonical multipotent state of MAF and guided cell fate change.

In addition, gene enrichment analysis highlighted the ribosome pathway among upregulated genes, which suggested a direct role of ribosomal activity for influencing cell fate change. This finding hinted at a potential mechanism where ribosomal components or associated factors acted as mirrored multipotency induction factors to modulate gene expression patterns. Further investigation is required to understand the precise molecular pathways involved.

In the beginning, RICs-MAF were tested for their differentiation capability by placing them in suitable conditions. It was well established that ribosome can induce stemness markers in RICs-HDF, which can be differentiated into three germ-layer-derived cells in vitro [13]. At this stage, we observed that RICs-MAF could efficiently be differentiated into adipocytes, osteoblasts, and chondrocytes. Moreover, cross-lineage differentiation to neural cells were also observed. However, this study experienced substantial differences from our previous studies, where the differentiation of RICs was achieved through typical multipotent cell phase. We noticed RICs-MAF cells were converted from one lineage to another while they did not follow the typical multipotent stage. This phenomenon fits with “cell plasticity” which is denoted as the morphological and molecular differentiation of both initial and final cells by the emergence of cellular intermediate states [3]. It differs from the concept of “dedifferentiation” as it states fully differentiated cells revert to a less differentiated (and proliferative) stage within the same lineage, without reaching pluripotency and “transdifferentiation” that states the direct conversion of a fully differentiated cells to another cell from distinct lineage, without passing through a pluripotent intermediate [35].

Lineage reprogramming has been affected by cellular phenotype, cell adhesion, and the factors that induce changes for acquiring a new cellular identity [23,42]. Fibroblasts retain a heterogenous transcriptome originating from their embryonic tissue sources and these transcriptional signatures possess direct translational consequences [43]. We hypothesized that such heterogeneity of adult fibroblast cells might be reasonable for acquiring rare multipotency during lineage conversion. Epigenetic changes that occurred during the maturation of fibroblasts with the remodeling of the chromatin landscape between MEF and MAF might have unexplored consequences during ribosome-induced plasticity. Considering the RT-PCR data, we speculated that RICs-MAF experienced an immature multipotent stage and converted to other cells when they were cultured in respective culture media. This assumption was supported by the reduction of fibroblast marker vimentin and fibroblast surface proteins in RICs-MAF, which suggested their conversion to another germ-layer-derived cell type.

Sequentially, we observed visually different expression patterns of vimentin in all the differentiated cells we have acquired. Fibroblasts are usually recognized as flat, spindle-shape cells, having a branched framework of cytoskeletal proteins, i.e., actin, vimentin, etc. Such expressions are not concentrated in the compact cytoplasm around the nucleus, while these proteins form a fibrous network among them [24,44]. The cell shape is observed as a filamentous network through the expression of cytoskeletal proteins, and changes in cell shape can have a major impact on cell fate decisions [26,27,45,46]. Our immunostaining data revealed RICs-MAF have rearranged their filament network, while they lost fibroblast characteristics and acquired a new pattern. These patterns are unique from the destined cell type and parental fibroblast. Based on this patterning, it is likely that RICs-MAF may reacquire cytoskeletal proteins that need to form the network de novo. We hypothesized that this cytoskeleton reorganization may have guided RICs-MAF toward lineage commitment to acquire a differentiated phenotype.

Afterwards, we observed that RICs-MAF retained their cluster shape and expressed early senescence markers that exhibit cell differentiation ability. In line with our previous findings, RICs-MAF experienced a pre-senescence state which was reversed in the differentiation induction medium. Such a reversion led to the generation of proliferative differentiated somatic cells. This resembled an interesting fact which states that cell reprogramming culture conditions significantly induce the expression of senescence markers [47]. For example, the expression of OSKM factors could induce a robust senescence response in multiple tissues and a positive correlation was observed between the reprogrammed cell and induction of senescence [48]. Furthermore, cell cycle control genes like p16 play a significant role in lineage and differentiation of cells, such as p16 promoting erythroid differentiation in viable K562 cells [49]. Likewise, we observed ribosome incorporation induced cell cycle arrest in MAF. Then differentiation was triggered and RICs-MAF could proceed independently. Despite a rare multipotent cellular state, exogenous ribosome might have induced an asynchronous effect in MAF that became more felicitous for plasticity following a proliferation cease-like state. It should be noted that proliferation and cell fate reversal are independent processes, which may work synergistically to enhance cell plasticity [35].

Our earlier works have observed exogenous ribosome localization in cytoplasm and the nucleus of ribosome-incorporated cells through immunostaining using antibody specific to exogenous ribosome [13]. In this study, we have used tetra-(His)6-tagged ribosomes from *E. coli* JE28 bacteria, which can produce functional ribosome with a His-tag in the L-12 ribosomal protein [18]. The tagged ribosomes were traceable in RICs-MAF cells to confirm the localization of exogenous ribosome. Our results indicated that incorporated ribosomes have existed in the cytoplasmic regions and the nuclear regions of MAF simultaneously, similar to our previous observation [13]. Though we could detect the existence of bacterial ribosome in the nucleus and the cytoplasm of a fibroblast cell by His-tag expression, it is difficult to predict the consequences of this incorporated ribosome. It will be intriguing to know whether exogenous ribosome maintained its structure or dissociated into protein sub-units (maybe single proteins) during plasticity induction. Currently, our group is working to find ribosomal protein(s) indispensable for multipotency induction that may answer such queries.

In the first instance, we hypothesized that the amount of ribosome incorporated in the cells might affect their multipotency and differentiation ability [39]. Here, we compared the amount of ribosome with the time necessary for cluster formation. Our results suggested 50 µg ribosome incorporated on 1.0 × 10^5^ cells for 72 h as the optimum requirement of ribosome to induce cluster formation, that later proceed to plasticity. Cell cluster formation is considered a salient property of multipotency and we experienced ribosome-induced cell cluster formation in our former works [13,38]. However, the optimal amount of ribosome required to generate cell clusters and subsequent multipotent status was documented here for the first time. Compared to other methods, we observed that multipotency was achieved here within a short time, in a concentration-controllable way, without the repeated administration of external stimuli.

Subsequently, we conducted AP staining, which is considered a standard method for screening pluripotent cells from terminally differentiated cells (feeder cells). Alkaline phosphatase expression is significantly elevated in pluripotent cells, which easily distinguishes them from feeder cells like fibroblast cells [9]. We hypothesized that ribosome incorporation induced multipotency in MAF and positive AP staining will support this hypothesis. Our results showed RICs-MAF were positive for AP staining tested on D3 and D14 and we anticipated these cells might achieve multipotency. Later, we observed RICs-MAF followed a unique strategy of cell plasticity through an unconventional multipotent state.

For clinical application, there is a pressing need for a safe and efficient approach to redirect cell fate. Alternative methods for non-invasively acquired cells, i.e., Sendai virus vectors or the expression of synthetic modified mRNA have been discussed lately to deliver clinical benefits for their non-invasive, non-mutagenic features [50,51]. However, generating functional cells with respect to the epigenetic landscape and developmental plasticity remains a challenge for the promotion of lineage conversion. Considering this, we have established a technique that enabled the conversion of somatic cells to multipotent cells toward a desired lineage. Our technique obviates stringent biological containment required for virus-based approaches, avoids the risk of mutagenesis, and is suitable for cost-effective maintenance. Here, we have observed cell identity changes into a specific lineage only in vitro. We speculate in vitro studies can provide valuable insights into the molecular mechanisms as well as the pathway associated with cell fate and regeneration. We expect transcriptome analysis between MEF and MAF may answer the queries behind the canonical and non-canonical ribosome-mediated multipotency acquisition. Moreover, we need to study the epigenetic landscape featuring DNA methylation, histone-marks and chromatin structure aimed at ribosome-incorporated multipotency in healthy cells and cancer cells. Moreover, extensive in vivo investigations are required to assess its applicability.

Cellular plasticity evolved from incorporating exogenous ribosome to mouse adult fibroblasts is not only a cell culture artifact but has paved the way to uncover the novel functional role of prokaryotic ribosome in mammalian cell lineage conversion. Such a functional impact of ribosome in a cross-species context needs further study to clarify this undeciphered phenomenon.

## 5. Conclusions

The efficiency of multipotency to generate cells and fidelity to the natural cell types are supported by the intrinsic properties of the starting cells. Here, we investigated MAF as a prospective source of mature somatic cells which could have important implications for designing regenerative therapies. This study employed “ribosome” as the driving force to induce plasticity in MAF. Ribosome incorporation stimulated multipotency acquisition in this cell that triggered differentiation and converted this cell type to diverse lineages unrelated to their original source. We have observed the upregulation of several pluripotency-associated genes, i.e., Dnmt3l, Sox5, Tbx3, and Cdc73; however, there was no visible induction of familiar markers like Oct4, Nanog, Sox2, etc. as seen in earlier studies. This suggests a cell-type-independent response to ribosomal stimulation and underscores the complexity of cellular plasticity. The acquisition of this distinctive multipotent state during lineage conversion could lead to a better-suited model for presumed late-onset disease conditions. Further investigation of ribosome-mediated lineage conversion of somatic cells will provide better insights into such a unique model of lineage commitment and may enable us to understand the underlying mechanism.

## Data Availability

The data is available upon request to the corresponding author.

## Supplementary Materials

The following supporting information can be downloaded at: https://www.mdpi.com/article/10.3390/cells13131116/s1, Figure S1: Illustration of mouse adult fibroblasts cell preparation from mouse ear pinnae.; Figure S2: RT-PCR to check multi-lineage differentiation markers in RICs-MAF. RT-PCR data revealed altered expression of Fibroblast marker gene (FAP) in RICs-MAF than Control-MAF (D14). In addition, lineage marker gene expression was tested after differentiation (D21). Differentiated cells generated from RICs-MAF have moved considerably from their fibroblast origin observed through the expression of adipogenesis marker (Adiponectin), osteogenesis marker (Osteocalcin), chondrogenesis marker (Col10a1) while Control-MAF retained their fibroblast characteristics.; Figure S3: RT-PCR to check vimentin expression in RICs-MAF (D14), noticeable reduction was observed in RICs-MAF compared to Control-MAF.; Figure S4: Remodeling of actin in RICs-MAF differentiated cells. Negative control (A–C) and RICs-MAF differentiated cells of adipocytes (D), osteoblasts (E), and chondrocytes (F) have been stained with anti-Actin antibody. Through immunostaining, RICs-MAF showed altered expression of actin that differs from fibroblasts. Fluorescence microscopic images are shown (scale bar: 50 µm).; Figure S5: RT-PCR to check senescence markers in RICs-MAF. RT-PCR data revealed upregulation of p16 and p19 expression in RICs-MAF than Control-MAF (D14).; Figure S6: Immunostaining of stemness marker expressions in ribosome-incorporated MEF (RICs-MEF) as positive control. (A) Merged expression of Oct4, Nanog and His, (B) His, (C) Nanog, (D) Oct4 and (E) Hoechst 33342. Sample preparation and staining conditions were uniformly maintained in RICs-MAF (tested cell) and RICs-MEF (positive control).; Figure S7: Immunostaining of Mouse IgG isotype control and Rabbit IgG isotype control as negative control. (A, D) Mouse IgG with Alexa 488, (B, E) Mouse IgG with FITC and (C, F) Rabbit IgG with Alexa 647 have been tested in RICs-MAF. Sample preparation and staining conditions were uniformly maintained in all staining experiments.; Figure S8: RT-PCR to check stemness markers in RICs-MAF. These data revealed upregulated expression of pluripotency-associated genes Dnmt3l, Sox5, Tbx3 and Cdc73 while downregulated expression of the fibroblast marker gene (FAP) in RICs-MAF than Control-MAF (D14). It showed RICs-MAF have acquired multipotency after ribosome incorporation, while Control-MAF retained their fibroblast characteristics.; Supplementary Figure S9: Full membrane of Western Blot images, details of each blot have been mentioned earlier.; Supplementary Figure S10: Uncropped images of Gel Electrophoresis after RT-PCR.; Supplementary Figure S11: Raw images of RICs-MAF presented in Figure 1H, I.; Supplementary Video S12: Live imaging of Control-MAF cells (24 h).; Supplementary Video S13: Live imaging of ribosome-incorporated MAF cells (24 h).; Supplementary Video S14: 10 µm section-Z-stack imaging of RICs-MAF (6 frames).; Supplementary Video S15: Zoomed central region-Z-stack imaging of RICs-MAF (14 frames).; Supplementary Table S1: RT-PCR Primers.; Supplementary Table S2: Antibodies used in Immunocytochemistry.; Supplementary Table S3: Antibodies used in Western blotting.; Supplementary Table S4: Statistical analysis of vimentin expression in Western blotting.; Supplementary Table S5: qRT-PCR data analysis for stemness markers.; Supplementary Table S6: RNA-sequencing analysis by Limma-voom tool.; Supplementary Table S7: RNA- Sequencing-Significant DEG analysis by Limma-voom tool for proinflammatory genes.
