# Supplementary material for: Bacterial Ribosomes Induce Plasticity in Mouse Adult Fibroblasts
Source: Cells. 2024 Jun 27;13(13):1116. doi: 10.3390/cells13131116 (PMC11240311; doi:10.3390/cells13131116)
Supplement: Supplementary file 1 [file cells-13-01116-s001.zip › Table S1. RT-PCR Primers.pdf]

Supplementary Table S1: RT-PCR Primers

No.	Gene	Forward	Reverse
1.	Fibroblast Activation protein (FAP)	CATGGGTGTCCAGTGAACGA	ACACACTTCTTGCTCGGAGG
2.	Adiponectin	GTCTGGCTCCAGGTGTATGG	GCGAATATTGTGAAGCCCCC
3.	Osteocalcin	GGTAGGAAGGAGTTGTGCTGG	AGATGCGTTTGTAGGCGGTC
4.	Col10a1	CCAGGCGCTAAAGGTGAGAT	GGTCGTAATGCTGCTGCCTA
5.	Gapdh	CAACTCACTCAAGATTGTCAGCA	GGCATGGACTGTGGTCATGA
6.	Sox5	CCCGTGATCCAGAGCACTTAC	CCGCAATGTGGTTTTCGCT
7.	Dnmt3l	GCTCTTGTGATCGCTGTCC	TCACAGCCTCTGTCTGAAGG
8.	Cdc73	AGATTGGCAGCTCGTTTGGA	CAATGTAGGATCCACGGGGG
9.	Tbx3	CAAGCGGGGTACAGAGATGG	GCCAGTGTCTCGAAAACCCT
10.	p16	GTGTGCATGACGTGCGGG	GCAGTTCGAATCTGCACCGTAG
11.	p19	GCTCTGGCTTTCGTGAACATG	TCGAATCTGCACCGTAGTTGAG
12.	Vimentin	GCCTGCAGGATGAGATCCAA	AAAAGGTTGGCAGAGGCAGA
