# Supplementary material for: Bacterial Ribosomes Induce Plasticity in Mouse Adult Fibroblasts
Source: Cells. 2024 Jun 27;13(13):1116. doi: 10.3390/cells13131116 (PMC11240311; doi:10.3390/cells13131116)
Supplement: Supplementary file 1 [file cells-13-01116-s001.zip › Table S2. Antibodies used in Immunocytochemistry.pdf]

Supplementary Table S2: Antibodies used in Immunocytochemistry

Primary Antibody		Host	Dilution	Company
1.	anti-6X-His	Rabbit	1:200	Abcam
2.	anti-Oct4	Rabbit	1:400	Abcam
3.	anti-Nanog	Rat	1:400	Invitrogen
4.	anti-Sox2	Rabbit	1:400	Abcam
5.	anti-Vimentin	Chick	1:400	Millipore
6.	Anti-Actin	Mouse	1:200	MP Biomedicals
7.	Anti-Runx	Mouse	1:50	Santa Cruz Biotechnology
8.	anti-β-tubulin (Tuj1)	Mouse	1:400	R&D systems
9.	anti-NG2	Rabbit	1:400	Millipore
10.	anti-GFAP	Rabbit	1:400	Dako
11.	anti-Mouse IgG Isotype Control	Mouse	1:400	Invitrogen
12.	anti-Rabbit IgG Isotype Control	Rabbit	1:400	Invitrogen
Secondary Antibody				
11.	anti-Mouse IgG conjugated with Fluorescein isothiocyanate (FITC)	-	1:800	Jackson ImmunoResearch
12.	anti-Mouse IgG conjugated with Alexa 488	-	1:800	Abcam
13.	anti-Rabbit IgG conjugated with Alexa 647	-	1:800	Abcam
14.	anti-Rat IgG conjugated with Cyanine-3 (cy3)	-	1:800	Jackson ImmunoResearch
15.	anti-Chick IgG conjugated with Alexa 488	-	1:800	Abcam
