# Supplementary material for: Bacterial Ribosomes Induce Plasticity in Mouse Adult Fibroblasts
Source: Cells. 2024 Jun 27;13(13):1116. doi: 10.3390/cells13131116 (PMC11240311; doi:10.3390/cells13131116)
Supplement: Supplementary file 1 [file cells-13-01116-s001.zip › Table S3. Antibodies used in Western Blotting.pdf]

Supplementary Table S3. Antibodies used in Western Blotting

Primary Antibody		Host	Dilution	Company
1	anti-6X-His	Rabbit	1:1000	Abcam
2.	anti-Actin	Mouse	1:2000	MP Biomedicals
3.	anti-P16	Rabbit	1:1000	Abcam
4.	anti-P19	Rabbit	1:1000	Abcam
5.	anti-Vimentin	Chick	1:1000	Millipore
Secondary Antibody				
7.	anti-Rabbit IgG HRP	-	1:10000	Jackson ImmunoResearch
8.	anti-Mouse IgG HRP	-	1:10000	Jackson ImmunoResearch
9.	Anti-Chick IgG HRP	-	1:10000	Abcam
