# Supplementary figures and images for: Bacterial Ribosomes Induce Plasticity in Mouse Adult Fibroblasts
Source: Cells. 2024 Jun 27;13(13):1116. doi: 10.3390/cells13131116 (PMC11240311; doi:10.3390/cells13131116)

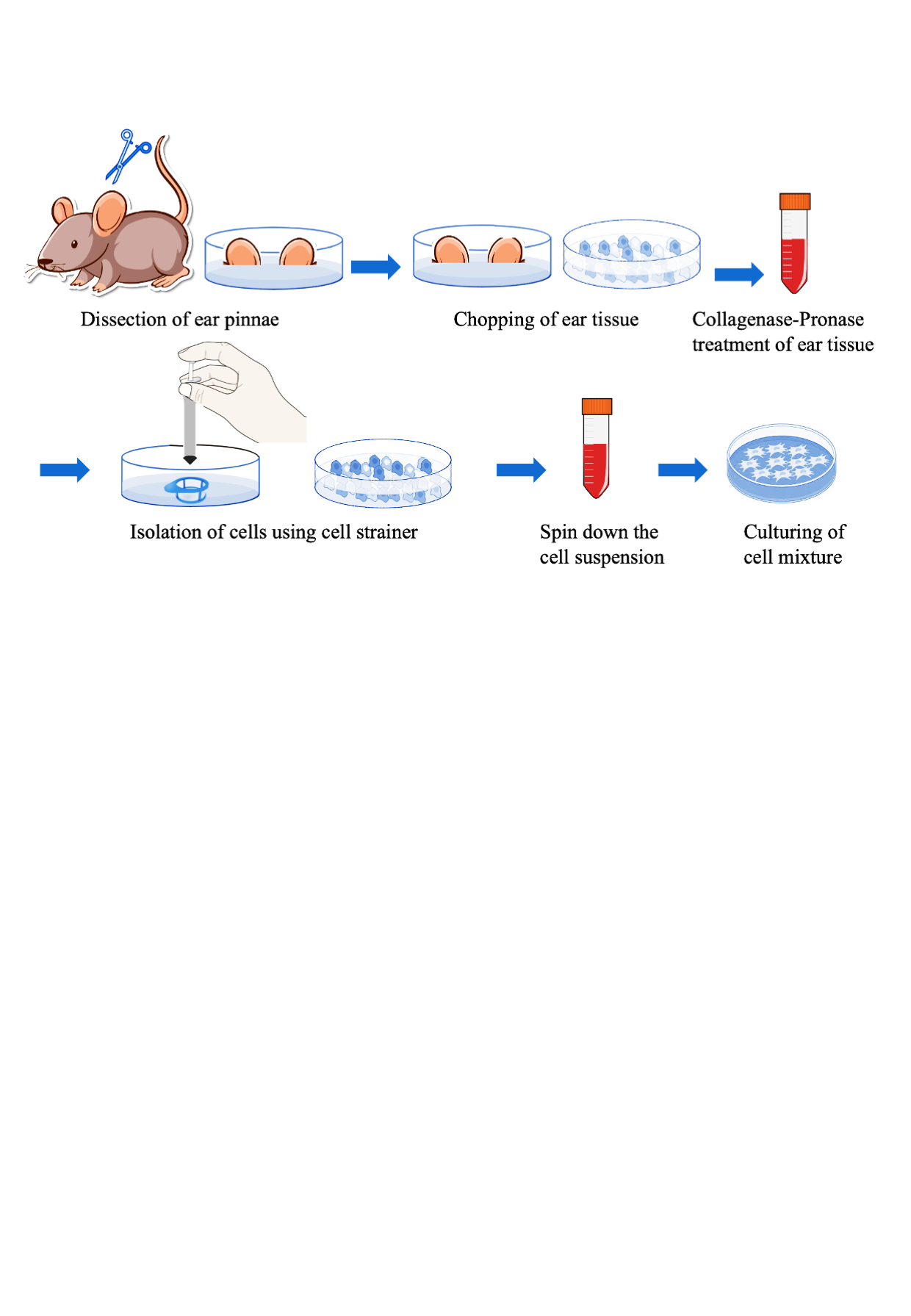

Supplement: Supplementary file 1 [file cells-13-01116-s001.zip › Figure S1.tiff]

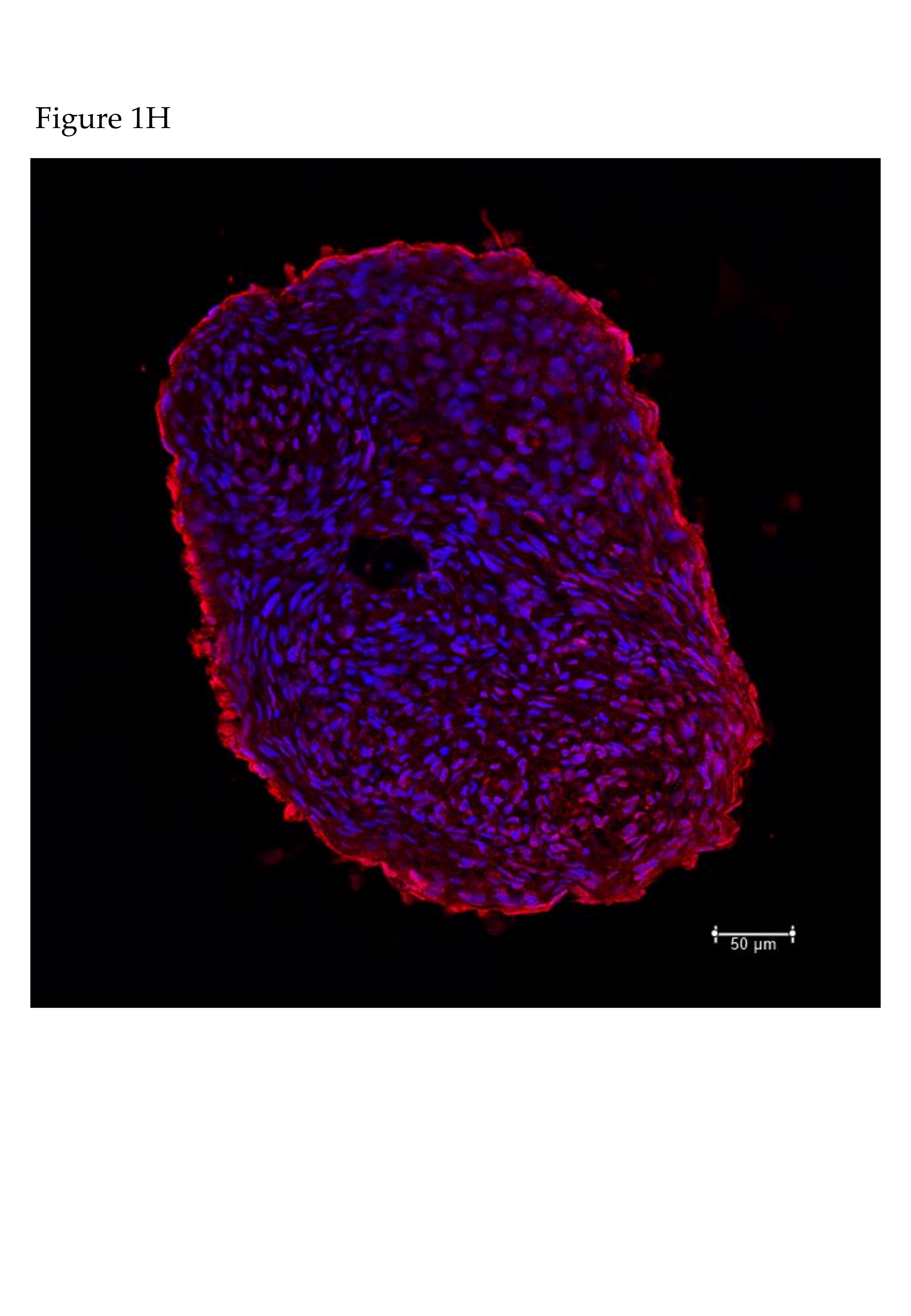

Supplement: Supplementary file 1 [file cells-13-01116-s001.zip › Figure S11.tiff]

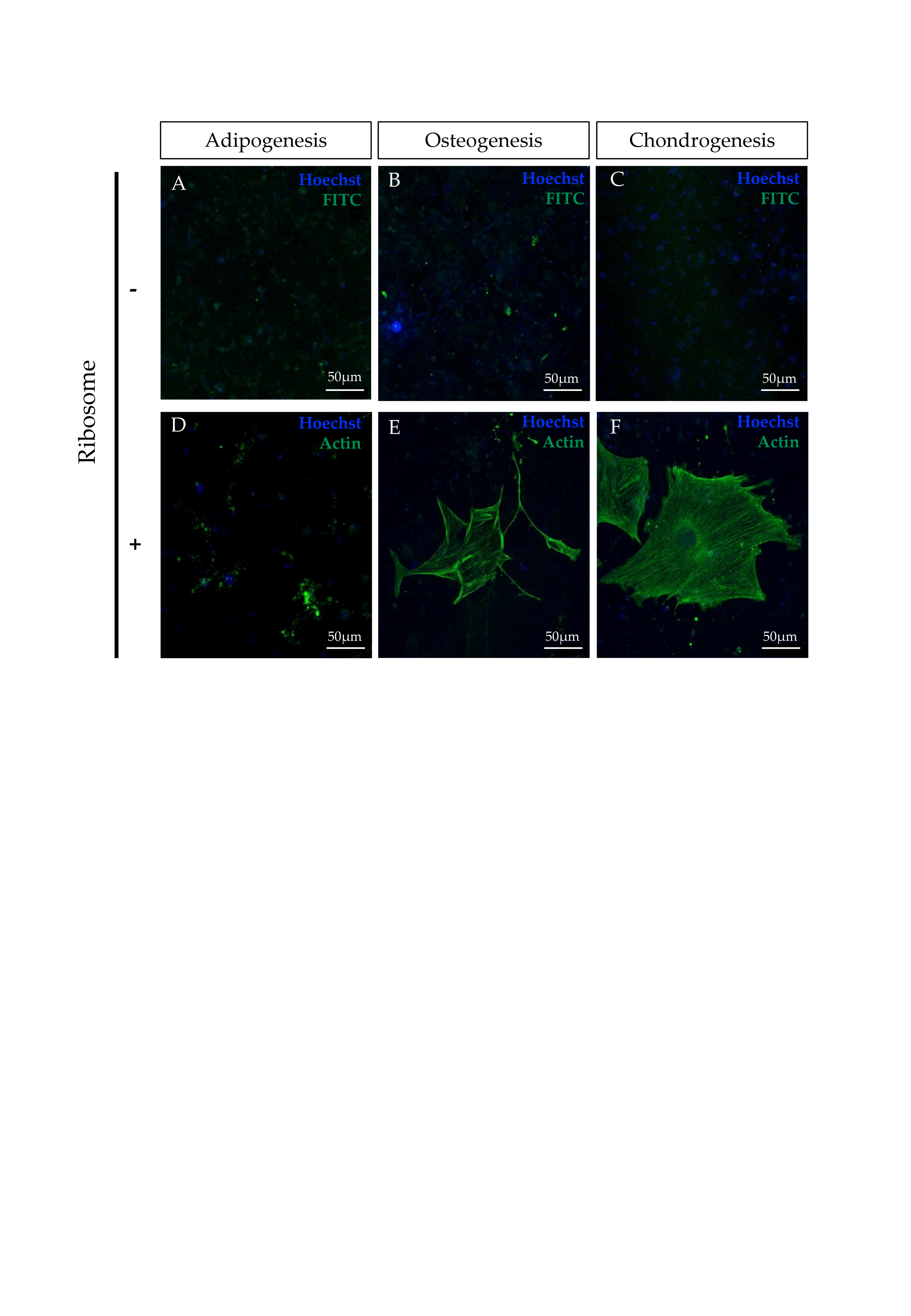

Supplement: Supplementary file 1 [file cells-13-01116-s001.zip › Figure S4.tiff]

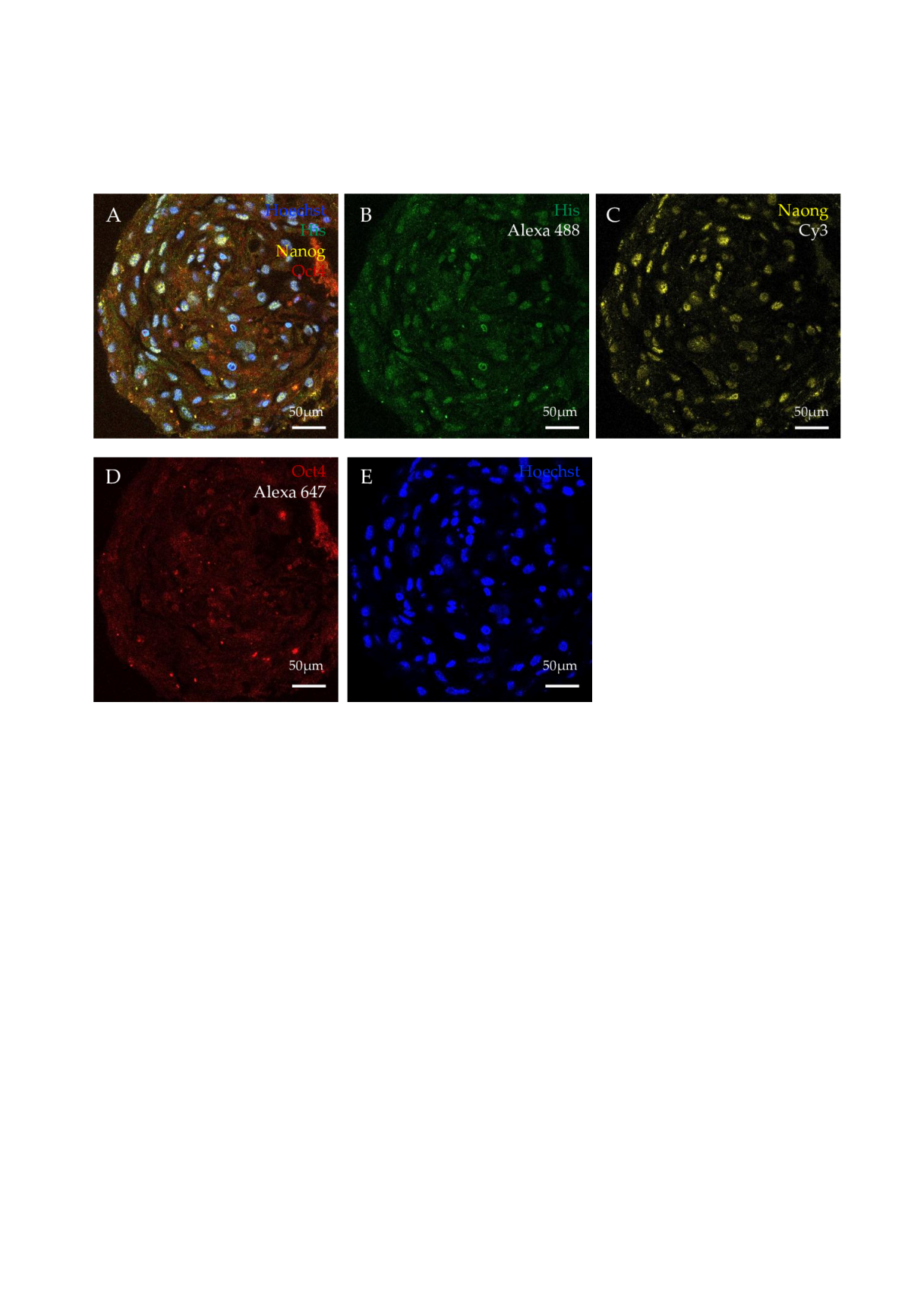

Supplement: Supplementary file 1 [file cells-13-01116-s001.zip › Figure S6.tiff]

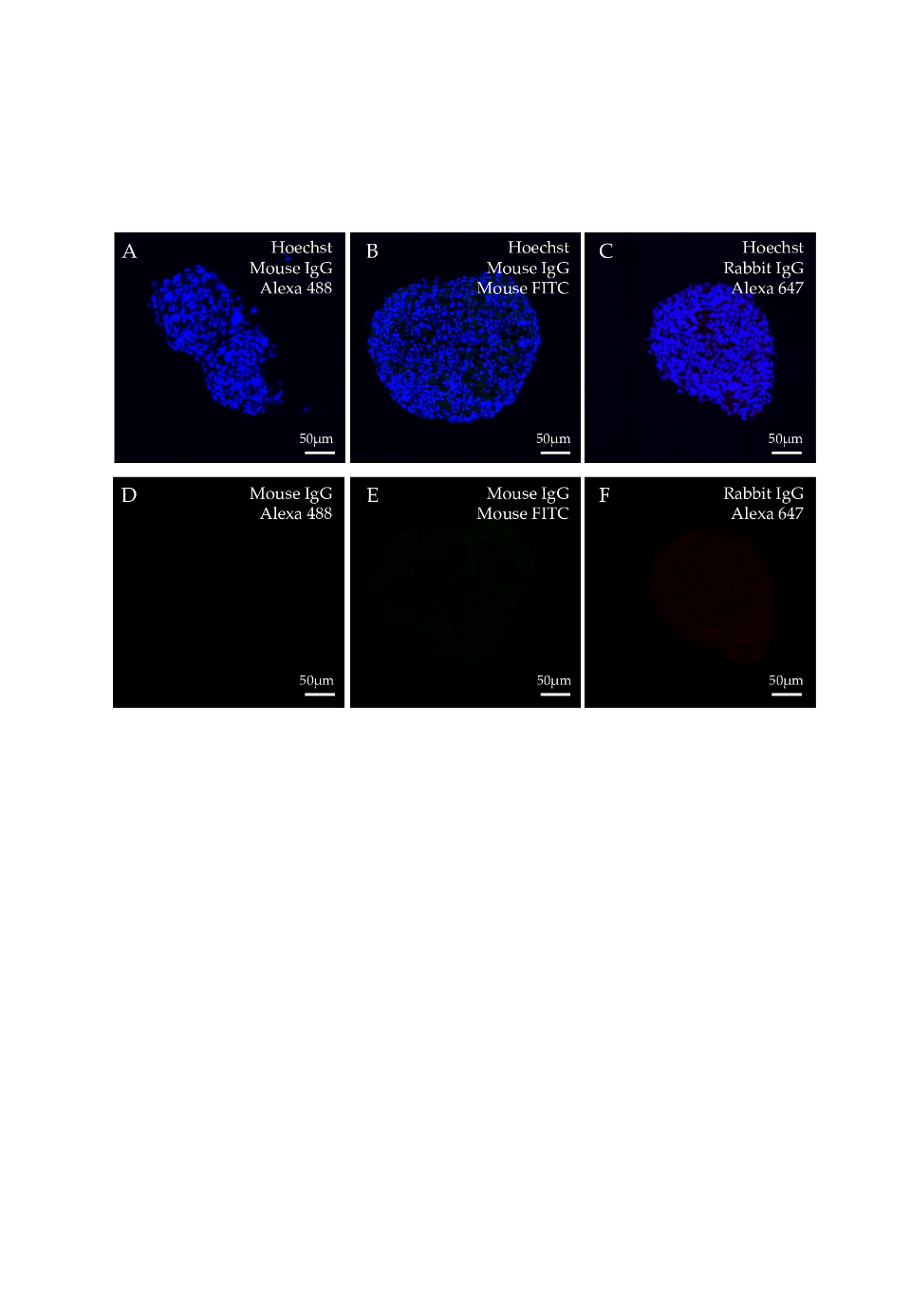

Supplement: Supplementary file 1 [file cells-13-01116-s001.zip › Figure S7.tiff]
